# Evaluation of the intraocular total IgE level and its ratio with serum IgE level for the diagnosis of ocular toxocariasis in children and adults: a retrospective comparative study

**DOI:** 10.1186/s12886-025-04252-z

**Published:** 2025-07-28

**Authors:** Shuang Zhang, Li Chen, Xiaofeng Hu, Hui Wang, Jing Feng, Yong Tao

**Affiliations:** Department of ophthalmology, Beijing Chao-Yang Hospital, Capital Medical University, No. 8 Gongtinan Road, Chaoyang District, Beijing, 100020 People’s Republic of China

**Keywords:** Ocular toxocariasis, Intraocular fluid, IgE, Diagnosis

## Abstract

**Background:**

Ocular Toxocariasis (OT) is characterized by complicated clinical manifestations, which makes it difficult to diagnose. In this study, we mainly evaluated the diagnostic values of intraocular fluid (IF) total IgE level and IF/Serum IgE ratio in OT and compared the differences between child and adult patients.

**Methods:**

76 patients with 52 children (< 16 years) and 24 adults of clinically-diagnosed OT and 64 patients with non-OT uveitis were enrolled in this study. The paired IF and serum samples were collected for total IgE, total IgG and specific IgG testing. The IF IgE levels and IF/Serum IgE ratios were compared between child and adult patients. The area under the curve (AUC) was generated to assess the diagnostic performances of the above indicators.

**Results:**

The IF IgE level was significantly higher in child patients than in adult patients (1671.79 ± 1425.97 versus 784.44 ± 544.73 ng/ml, *p* = 0.015). Besides, the IF/Serum IgE ratio showed similar trend with 24.02 ± 6.10 in children and 7.68 ± 5.05 in adults (*p* = 0.001). The best cutoff value of the IF IgE concentration was 10.65 ng/ml, yielding 77.6% (95%CI, Confidence Interval, 66.4–86.1%) sensitivity and 98.4% (95%CI, 90.5–99.9%) specificity. The IF IgE level showed lower AUC than that of IF specific IgG level (0.925 versus 0.975, *p* = 0.037). However, the AUC of IF/Serum IgE ratio for OT was significantly higher than that of GWC (0.934 versus 0.527, *p* < 0.0001). The best cutoff value of IF/Serum IgE ratio was 0.48 with the sensitivity of 78.4% (95%CI, 67.0-86.8%) and specificity of 98.4% (95%CI, 90.5–99.9%). For the diagnosis of child OT, the cutoff value of IF IgE level of child OT was 13.10 ng/ml with the high sensitivity of 82.7% (95%CI, 69.2–91.3%) while the best cutoff value of 10.65 ng/ml for adult OT yielded only 66.7% (95%CI, 44.7–83.6%) sensitivity. However, the sensitivity of IF/Serum IgE ratio for child OT (86.3%, 95%CI, 73.1–93.8%) was close to that for adult OT (87.0%, 95%CI, 65.3–96.6%).

**Conclusions:**

Both IF IgE level and IF/Serum IgE ratio demonstrated good diagnostic performance for ocular toxocariasis and higher sensitivity of IF IgE level was noted for the diagnosis of child OT.

## Background

Human toxocariasis is a worldwide zoonotic helminth infection caused by parasitic nematodes including Toxocara canis and Toxocara cati. These parasites are usually directly transmitted to the human host via the faecal-oral route and can cause various clinical forms of toxocariasis such as ocular larva migrans, visceral larva migrans, covert or common toxocariasis, and neurotoxocariasis [1]. Ocular toxocariasis (OT) is a vision-threatening disease prevalent in poor-hygiene regions or rural areas and occurs primarily in children [2]. The gold standard for OT diagnosis is histological detection of Toxocara larvae or their fragments from biopsy specimens, which is actually hard to achieve in clinical practice. Therefore, the diagnosis of OT usually relies on the history of exposure to dogs and cats [3, 4], ocular manifestations and laboratory examinations involving the detection of Toxocara antibody in serum and intraocular fluid (IF) [5, 6]. The clinical presentations of OT are complicated and can mimic other diseases such as retinoblastoma, ocular toxoplasmosis and other cause of uveitis which can result in retinal granuloma and vitritis. Several studies have confirmed the diagnostic value of IF specific IgG and Goldmann-Witmer coefficient (GWC) for OT, which represents the local production of the antibody during the disease [5–8]. However, suspected OT patients with typical granulomatous uveitis and negative results of IF specific IgG detection could be noted in several cases [5–7]. Therefore, it’ s meaningful to evaluate other indicators to compensate the current diagnosis standard.

The production of IgE is the reaction of Th2 immune response and represents not only allergy but also infection of helminths [9, 10]. Anti-parasite IgE has been associated with immunity against a range of helminth infections and it is widely-accepted that IgE and its receptors evolved to help counter parasites [11]. Although there are many studies concerning the prevalence and diagnostic performance of IF specific IgG for OT, little is known about the diagnostic value of IF total IgE for OT. In this study, we mainly focused on the diagnostic values of IF total IgE and its ratio with serum IgE and first confirmed the diagnostic cutoff values of the above two indicators for OT. Besides, we also compared the differences of the concentrations of total IgE in IF and its ratios with serum IgE between child and adult OT patients and investigated the diagnostic performances of the above two indicators in child and adult OT respectively.

## Methods

### Subjects and controls enrolled

From March 2018 to November 2021, 76 consecutive patients with clinically-diagnosed OT at Beijing Chao-Yang Hospital were included in this study. The inclusion criteria for OT were as follows: unilateral eye involvement; clinical features of OT, including the presence of typical peripheral granuloma, posterior pole granuloma or vitreous opacity with unknown reasons; definite contact history with cats and dogs (or living in rural area); Experience of OT-related vitrectomy or cataract surgery for IF collection and paired serum samples collected simultaneously. The exclusion criteria included other intraocular granulomatous diseases except OT, sarcoidosis, tuberculosis, and fungal endophthalmitis. The age of the patients ranged from 3 years to 77 years old; Patients with age < 16 years were considered as children and those with age ≥ 16 years were categorized as adults. 64 patients with non-OT uveitis during the same period were enrolled as normal controls. The IF samples and the paired serum samples were also obtained from them.

We performed this study in accordance with the Declaration of Helsinki, and we received approval from the ethics committee of the Beijing Chaoyang Hospital, Capital Medical University (No. 2018-4-3-3). Written informed consent was obtained from each patient or guardian of pediatric parents with thorough explanation of the purpose including additional ocular fluid analysis and potential adverse effects of the procedure.

### Sample acquisition and handling

IF specimens including 28 aqueous humor (AH) samples and 48 vitreous humor (VH) samples of OT patients were obtained and paired serum specimens were collected as well. Undiluted aqueous samples of 0.3 mL or undiluted vitreous samples of 0.5 mL were aspirated and placed in a sterilized plastic corning (2 mL; Corning, Inc, Troy, MI) at the beginning of pars plana vitrectomy performed for OT-related complications including epiretinal membrane, retinal detachment or severe vitreous opacity. Besides, 0.3 mL of the AH was also extracted during the cataract surgery or intravitreal injection for macular edema. All samples were placed on ice immediately and stored at −80 °C for further measurement including total IgE, total IgG and specific anti-T. canis IgG testing.

### Total IgE level testing

Total IgE level was determined by the cytometric bead array (CBA)(Human IgE Flex Set kit, BD Biosciences, Cat.No.:558682). The assays were performed according to the manufacturer’s instructions. Briefly, Add 50 µL of each AH or VH sample to the appropriate assay tubes; Add 50 µL of the Capture Beads coated with IgE antibodies to each assay tube; Incubate the tubes for 1 h at room temperature and then add 50 µL of the mixed PE Detection Reagent to each assay tube; Wash the beads with wash buffer, and add 300 µL of Wash Buffer to each assay tube. The determination was performed with FACS Canto II flow cytometer (BD Biosciences).

### Anti-T.canis IgG level testing and calculation of GWC (Goldmann-Witmer coefficient)

The determination of anti-T.canis IgG levels in IF and serum samples is based on the Enzyme-linked Immunosorbent Assay (ELISA) technique, with Toxocaracanis IgG ELISA kit (RE58721, IBL International, Inc., Germany). Total IgG levels were determined by the cytometric bead array (CBA) (Human Total IgG Flex Set kit, BD Biosciences, Cat.No.:558679). The assays were performed according to the manufacturer’s instructions. The GWC was calculated as ([specific IgG in IF/specific IgG in serum]/[total IgG in IF/total IgG in serum]).

### Statistical analysis

In this study, all the statistical analyses were performed using Statistical Package for the Social Sciences software, version 26.0 (SPSS Inc., Chicago, IL). Mann-Whitney U test was applied to compare the serum and IF IgE concentrations between different groups. α value was adjusted by Holm-Bonferroni for multiple comparisons between different subgroup tests. For multiple comparisons, *p* values of < adjusted α were considered to indicate statistically significant differences while *p* values of < 0.05 were considered to show statistically significant differences for other tests. Chi-square test was used to compare binary categorical variables. The maximum Youden Index was calculated to determine the best cutoff values of different indicators. DeLong’s test was employed to compare the AUCs of different indicators.

## Results

### Demographic and clinical features

The demographic and clinical features of the patients with OT are shown in Table 1. In total, 76 patients with clinical-diagnosed OT were enrolled in this study, including 52 children and 24 adults. There were more male patients in children (63.5%) than in adults (33.3%) with significant difference (*p* = 0.014). The eye genders of child and adult patients were similar. The percentage of patients with contact history with dogs and cats was 75% and the other 25% patients mostly lived in rural area. 28 AH specimens and 48 VH specimens were collected for antibody detection in total, with no significant difference between child and adult patients. Besides, the mean duration from disease onset to intraocular fluid testing was 11.01 ± 13.05 months. In terms of clinical characteristics, the LogMAR visual acuity of children patients was significantly higher than that in adults (1.13 ± 0.71 versus 0.64 ± 0.53, *p* = 0.001). The mean intraocular pressure of children and adult patients was close with *p* = 0.415. And 38.2% of the OT patients presented with retinal detachment with 42.3% in children and 29.2% in adults.

**Table 1:** Demographic and clinical features of the patients with ocular toxocariasis in this study

	Total	Children	Adults	*p* value
Total (*n*, %)	76(100%)	52(68.4%)	24(31.6%)	-
Gender (*n*, %)				
Male	41(53.9%)	33(63.5%)	8(33.3%)	0.014^a^
Female	35(46.1%)	19(36.5%)	16(66.7%)	
Eye gender (*n*, %)				
Right	46(60.5%)	32(58.3%)	14(61.5%)	0.790^a^
Left	30(39.5%)	20(41.7%)	10(38.5%)	
Contact history with dogs and cats (*n*, %)				
Yes	57(75%)	40(76.9%)	17(70.8%)	0.569^a^
No	19(25%)	12(23.1%)	7(29.2%)	
Type of intraocular fluid tested (*n*, %)				
Aqueous humor	28(36.8%)	18(34.6%)	10(41.7%)	0.554^a^
Vitreous humor	48(63.2%)	34(65.4%)	14(58.3%)	
Retinal detachment (*n*, %)				
Yes	29(38.2%)	22(42.3%)	7(29.2%)	0.273^a^
No	47(61.8%)	30(57.7%)	17(70.8%)	
Visual Acuity (logMAR) Mean±SD	0.99±0.69	1.13±0.71	0.64±0.53	0.001^b^
Intraocular pressure (mmHg) Mean±SD	15.33±4.01	15.69±4.61	14.57±2.12	0.415^b^
Duration from disease onset to intraocular fluid testing (month) Mean±SD	11.01±13.05	11.82±14.33	9.20±9.67	0.621^b^

Children, Age＜16 years old; Adult, Age≥16 years old

*SD* Standard deviation

^a^the *p* value was calculated by chi-square test

^b^the *p* value was calculated by Mann-Whitney U test

### Serum and intraocular fluid IgE level testing

**Fig. 1:**
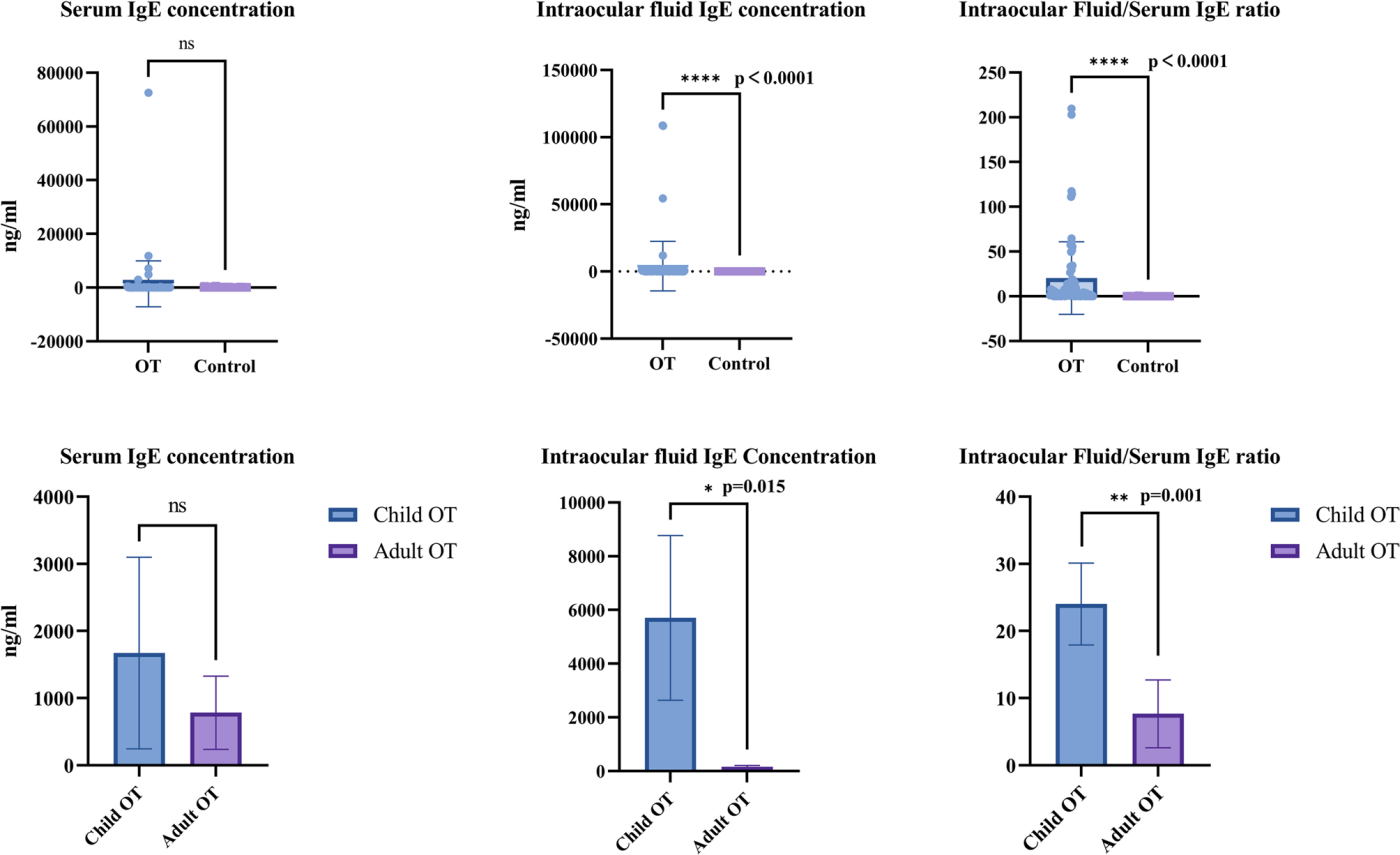
Comparisons of intraocular fluid IgE concentrations and intraocular fluid/serum IgE ratios between different groups. OT, ocular toxocariasis; Mann-Whitney U test was applied to compare the intraocular fluid IgE concentrations and intraocular fluid/serum IgE ratios between different groups. Holm-Bonferroni was employed to adjust α value for multiple comparisons. *p* values < adjusted α was considered as significantly different

The results of serum and IF IgE concentrations between different groups are displayed in Fig. 1 and Table 2. As Fig. 1 showed, there was no significant difference of serum IgE levels between OT and control group. On the contrary, the IgE concentrations in IF was significantly higher in OT patients than in control group (1395.99 ± 994.97 ng/ml versus 102.48 ± 16.56 ng/ml, *p* < 0.001). Besides, the IF/Serum IgE ratio of OT patients (18.94 ± 4.55) was higher than that of control group (0.05 ± 0.02) with *p* < 0.001. As for the difference of IgE level between child and adult patients, child OT patients had higher IF IgE concentration than adult patients (1671.79 ± 1425.97 ng/ml versus 784.44 ± 544.73 ng/ml, *p* = 0.015 < adjusted α = 0.025). In addition, the IF/Serum IgE ratio showed similar trend with 24.02 ± 6.10 in children and 7.68 ± 5.05 in adults (*p* = 0.001 < adjusted α = 0.016). However, there was no significant difference of serum IgE levels between child and adult patients.

**Table 2:** The concentrations of serum IgE and intraocular fluid IgE and IF/Serum IgE ratios in different groups of patients with ocular toxocariasis

	Serum IgE level (ng/ml)	Intraocular fluid IgE level (ng/ml)	IF/Serum IgE ratio
Total (Mean±SEM)	1395.99±994.97	3954.82±2114.83	18.94±4.55
Gender (Mean±SEM)			
Male	610.73±353.60	4273.68±2925.49	18.39±4.57
Female	2271.00±2072.55	3581.30±3101.70	19.57±8.25
*p* value	0.649	0.026	0.158
α adjusted	0.050	0.016	0.025
Eye gender (Mean±SEM)			
Right	2038.71±1652.84	6401.08±3460.95	17.75±5.58
Left	453.34±392.73	203.90±38.33	20.70±7.80
*p* value	0.664	0.386	0.939
α adjusted	0.025	0.016	0.050
Type of intraocular fluid tested (Mean±SEM)			
Aqueous humor	668.43±465.91	572.67±420.29	15.54±7.87
Vitreous humor	1813.96±1546.81	5927.74±3319.08	20.90±5.60
*p* value	0.193	0.030	0.128
α adjusted	0.050	0.016	0.025
Retinal detachment (Mean±SEM)			
Yes	3453.37±2705.08	7691.14±5187.59	19.15±8.11
No	214.09±119.65	1649.43±1175.41	18.82±5.51
*p* value	0.271	0.604	0.740
α adjusted	0.016	0.025	0.050
Duration from disease onset to intraocular fluid testing (Mean±SEM)			
＜1 year	511.47±306.70	1510.25±1261.73	18.24±5.72
≥1 year	2912.12±2592.60	8402.73±5361.53	22.53±8.45
*p* value	0.260	0.663	0.857
α adjusted	0.016	0.025	0.050

The *p* value was determined by Mann-Whitney U test. α value was adjusted by Holm-Bonferroni for multiple comparisons. *IF*, intraocular fluid

Table 2 shows other variables that might have association with IgE concentration except the age category. The intraocular fluid IgE level was higher in male patients than in female patients (4273.68 ± 2925.49 ng/ml versus 3581.30 ± 3101.70 ng/ml, *p* = 0.026). However, the p value of 0.026> adjusted α = 0.016 demonstrated the difference was not significant. Likewise, the IgE concentration (5927.74 ± 3319.08 ng/ml) in VH was higher than that in AH (572.67 ± 420.29 ng/ml, *p* = 0.030). After comparing the p value with adjusted α (0.016), the difference was not significant. In addition, the other variables including eye gender, retinal detachment and duration from disease onset to intraocular fluid testing had no remarkable influence on the IgE level and IF/Serum ratio.

### The diagnostic value of intraocular IgE level and IF/Serum IgE ratio

**Fig. 2:**
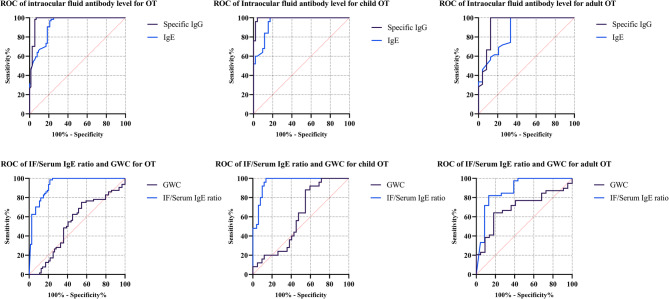
ROC curves of different indicators for the diagnosis of ocular toxocariasis, child and adult ocular toxocariasis. OT, ocular toxocariasis; IF, intraocular fluid

The diagnostic value of IF IgE level and IF/Serum IgE ratio for OT were analyzed by ROC curves and showed in Fig. 2. We compared the ROC curves of IF IgE level and IF/Serum IgE ratio with IF specific IgG level and GWC respectively. The best cutoff values and associated parameters are displayed in Table 3. According to the ROC curves in Fig. 2, the intraocular IgE level showed lower AUC than that of intraocular specific IgG level (*p* = 0.037). For all the OT patients, the AUC of IF IgE level was 0.925 (95%CI, Confidence Interval, 0.883–0.966) and AUC of IF IgG level was slightly higher with 0.975 (95%CI, 0.948-1.000). When the IgE concentration of 10.65 ng/ml was considered as test-positive, the sensitivity and specificity was 77.6% (95%CI, 66.4–86.1%) and 98.4% (95%CI, 90.5–99.9%) respectively. On top of that, the IF IgE level showed better diagnostic performance in child OT patients than in adult OT patients. For child OT patients, the cutoff value of IgE level was 13.10 ng/ml with the high sensitivity of 82.7% (95%CI, 69.2–91.3%) while the sensitivity with the cutoff value of 10.65ng/ml among adult patients was only 66.7% (95%CI, 44.7–83.6%). When comparing the diagnostic performances of IF IgE level and specific IgG level for child OT, we found the AUC of IF IgG level was also higher than that of IF IgE level (0.948 versus 0.994, *p* = 0.048).

**Table 3:** Cutoff values and associated parameters of different indicators for the prediction of ocular toxocariasis under different scopes of patients

Scope of patients	Type of indicator	Cutoff value	Sensitivity% (95%CI)	Specificity% (95%CI)	Youden Index	AUC (95%CI)	*p* value	Z value	*p* value of DeLong’s test
For the diagnosis of OT	IF IgE level (ng/ml)	10.65	77.6 (66.4,86.1)	98.4 (90.5,99.9)	0.761	0.925 (0.883,0.966)	<0.0001	-2.089	0.037
	IF IgG level (U/L)	1.01	93.2 (84.1,97.5)	100.0 (92.9,100.0)	0.932	0.975 (0.948,1.000)	<0.0001		
	IF/Serum IgE ratio	0.48	78.4 (67.0,86.8)	98.4 (90.5,99.9)	0.768	0.934 (0.894,0.974)	<0.0001	7.088	<0.0001
	GWC of IgG	40.20	45.3 (33.0,58.2)	75.0 (62.3,84.6)	0.203	0.527 (0.425,0.630)	0.594		
For the diagnosis of child OT	IF IgE (ng/ml)	13.10	82.7 (69.2,91.3)	100.0 (83.4,100.0)	0.827	0.948 (0.904,0.992)	<0.0001	-1.980	0.048
	IF IgG level (U/L)	1.02	95.9 (84.9,99.3)	100.0 (83.4,100.0)	0.959	0.994 (0.983,1.000)	<0.0001		
	IF/Serum IgE ratio	0.52	86.3 (73.1,93.8)	100.0 (83.4,100.0)	0.863	0.956 (0.915,0.998)	<0.0001	7.639	<0.0001
	GWC of IgG	48.43	35.7 (22.0,52.0)	76.0 (54.5,89.8)	0.117	0.413 (0.274,0.552)	0.218		
For diagnosis of adult OT	IF IgE (ng/ml)	10.65	66.7 (44.7,83.6)	100.0 (88.8, 100.0)	0.667	0.864 (0.770,0.959)	<0.0001	-1.164	0.295
	IF IgG level (U/L)	0.99	87.5 (66.5,96.7)	100.0 (88.8, 100.0)	0.875	0.934 (0.858,1.000)	<0.0001		
	IF/Serum IgE ratio	0.02	87.0 (65.3,96.6)	82.1 (65.9,91.9)	0.593	0.859 (0.814,0.976)	<0.0001	2.406	0.016
	GWC of IgG	23.49	81.8 (59.0,94.0)	64.1 (47.1,78.3)	0.365	0.698 0.564,0.832)	0.011		

The maximum Youden Index was calculated to determine the best cutoff values of different indicators

*OT* Ocular toxocariasis, *IF* Intraocular fluid, *GWC* Goldmann-Witmer coefficient, *AUC* The area under the curve

**Fig. 3:**
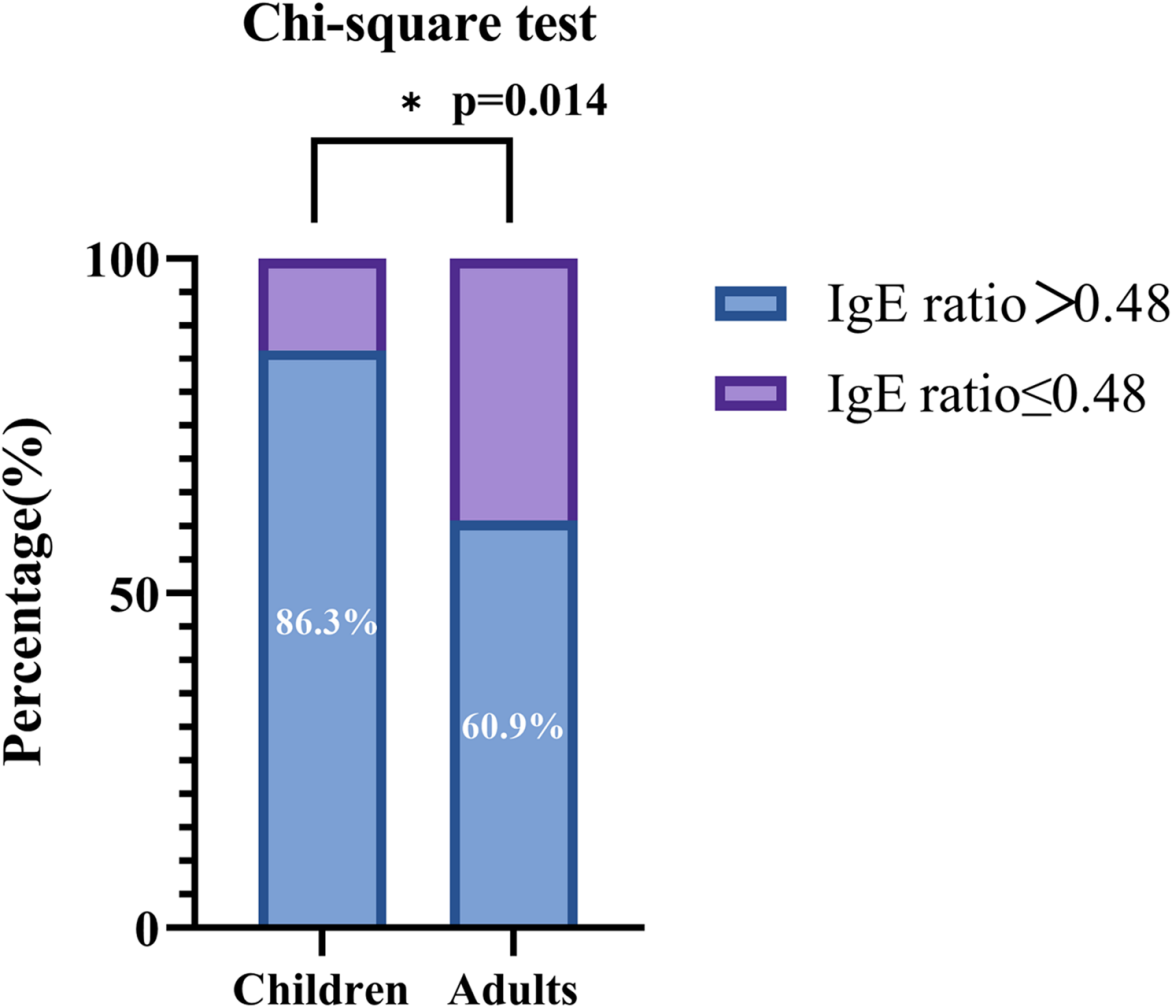
The percentages of patients with intraocular fluid/serum IgE ratio > 0.48 between different age groups

In terms of IF/Serum IgE ratio, the cutoff value was 0.48 for all OT patients yielding 78.4% (95%CI, 67.0-86.8%) sensitivity and 98.4% (95%CI, 90.5–99.9%) specificity. The percentage of patients with IF/Serum IgE ratio>0.48 was significantly higher in child patients (86.3%) than in adult patients (60.9%) with *p* = 0.014 (Fig. 3). The AUC of IF/Serum IgE ratio was significantly higher than that of GWC of IgG (0.934 versus 0.527, *p* < 0.0001). The diagnostic performance of plain GWC without IF IgG titer was limited with the sensitivity of 45.3% (95%CI, 33.0-58.2%) and specificity of 75% (95%CI, 62.3–84.6%). The IF/Serum IgE ratio showed both good diagnostic performances for child OT and adult OT. When IF/Serum IgE ratio>0.52 was defined as test-positive for children OT patients, the sensitivity was as high as 86.3% (95%CI, 73.1–93.8%) and the specificity was 100% (95%CI, 83.4–100.0%). Similarly, the sensitivity was 87.0% (95%CI, 65.3–96.6%) for adult OT with the low cut-off value of 0.02. Since the cutoff value was extremely low, the specificity (82.1%, 95%CI, 65.9–91.9%) was lower than that in child OT patients. Besides, for the diagnosis of OT both in children and adults, IF/Serum IgE ratio showed better performance than GWC of IgG (*p* < 0.05).

**Fig. 4:**
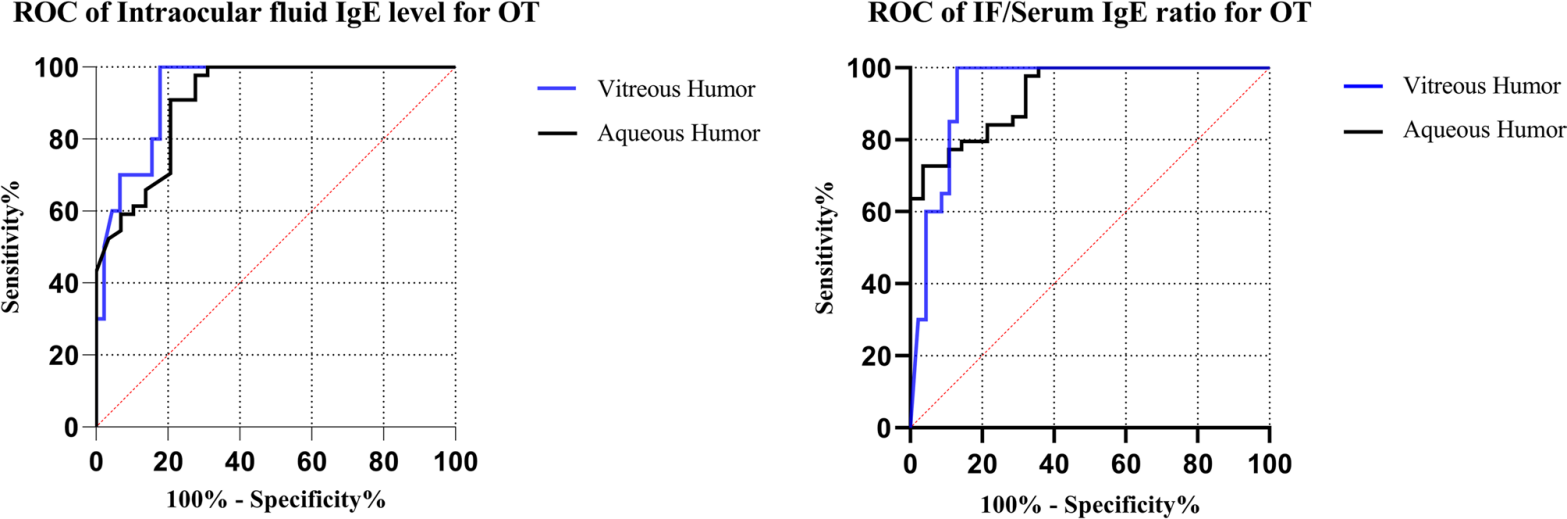
ROC curves of different indicators in AH and VH respectively for the diagnosis of ocular toxocariasis OT, ocular toxocariasis; IF, intraocular fluid

In addition, we also evaluated the diagnostic values of total IgE level and IF/Serum IgE ratio of AH and VH for OT respectively (Fig. 4). The associated parameters of ROC curves were displayed in Table 5. For both IF IgE level and IF/Serum IgE ratio, the AUCs of AH and VH showed no significant difference (*p* = 0.472 and *p* = 0.659). The cutoff value of IF IgE level in AH was 4.72 ng/ml, yielding 78.6% (95%CI, 58.5–91.0%) sensitivity and 90.9% (77.4–97.0%) specificity. The cutoff value of IF IgE level in VH was higher with 10.65 ng/ml, yielding higher sensitivity 83.3% (95%CI, 69.2–92.0%) and higher specificity 100% (80.0-100.0%). Similarly, higher cutoff value of IF/Serum IgE ratio was noted in VH than in AH (0.19 versus 0.02).

**Table 4:** Cutoff values and associated parameters of different indicators in aqueous humor and vitreous humor respectively for ocular toxocariasis

Type of indicator	Type of IF	Cutoff value	Sensitivity (95%CI)	Specificity (95%CI)	Youden Index	AUC (95%CI)	*p* value	Z value	*p* value of DeLong’s test
IF IgE level (ng/ml)	AH	4.72	78.6 (58.5,91.0)	90.9 (77.4,97.0)	0.695	0.906 (0.836,0.977)	<0.0001	-0.719	0.472
	VH	10.65	83.3 (69.2,92.0)	100 (80.0,100.0)	0.833	0.939 (0.886,991)	<0.0001		
IF/Serum IgE ratio	AH	0.02	92.6 (74.2,98.7)	77.3 (61.8,88.0)	0.699	0.934 (0.864,1.000)	<0.0001	-0.441	0.659
	VH	0.19	87.2 (73.6,94.7)	100 (80.0,100.0)	0.872	0.943(0.890, 0.995)	<0.0001		

The maximum Youden Index was calculated to determine the best cutoff values of different indicators

*OT* Ocular toxocariasis, *IF* Intraocular fluid, *AUC* The area under the curve, *AH* Aqueous humor, *VH* Vitreous humor

**Table 5:** Clinical and laboratory data of 9 patients with a negative result of intraocular specific IgG (≤ 1.8U/L) and a positive result of intraocular total IgE (> 10.65ng/ml) or if/serum IgE Raito (> 0.48)

Patient	Age	Gender (M, male; F, female)	Contact history with dogs and cats	Living in rural area	Duration from disease onset to IF testing (month)	Type of IF tested (AH, aqueous humor; VH, vitreous humor)	VA (LogMAR)	IOP	Clinical features (PG, peripheral granuloma; PPG, posterior pole granuloma)	RD	IF specific IgG (U/L)	Serum specific IgG (U/L)	IF total IgG (U/L)	Serum total IgG (U/L)	GWC	IF IgE (ng/ml)	Serum IgE (ng/ml)	IF/Serum IgE ratio
1	4	F	No	Yes	1	AH	1.3	17	PG	No	1.5	3.0	497.1	7050.0	7.1	33.3	1.0	33.3
2	6	F	Yes	-	48	VH	2.0	15	PPG	Yes	1.4	1.1	-	-	-	108420.4	72577.7	1.5
3	7	F	Yes	Yes	1.5	VH	2.3	13	PG	No	1,0	1.8	1914.9	17490.0	4.8	1.0	1.2	0.8
4	7	M	Yes	Yes	3	VH	1.0	17	PG	No	1.0	12.8	29.0	6675.0	18.7	13.9	67.3	0.2
5	8	F	No	Yes	6	AH	2.0	12	PG	Yes	1.1	2.7	149.4	13042.5	33.7	75.8	19.4	3.9
6	9	M	No	Yes	1	AH	0.7	11	PG	Yes	0.8	2.1	812.9	15855.0	7.7	5.7	7.3	0.8
7	23	F	Yes	-	6	AH	0.3	16	PG	No	1.0	6.0	79.5	11325.0	24.1	566.4	4911.0	0.1
8	29	F	Yes	-	24	VH	0.4	12	PG	No	1.2	2.7	1966.5	42000.0	9.8	1.0	0.5	2.0
9	37	M	Yes	Yes	3	AH	0.7	17	PG	Yes	0.7	2.9	297.8	8092.5	6.3	463.3	11829.8	0.0

*IF *Intraocular fluid, *GWC *Goldmann–Witmer coefficient, *VA*, Visual acuity, *IOP *Intraocular pressure, *RD *Retinal detachment

Based on the cutoff value of anti-Toxocara IgG confirmed by larger sample size (290 subjects), IF specific IgG ≤ 1.8U/L is considered negative [7]. Table 5 displays the clinical and laboratory data of 9 patients with a negative result of IF specific IgG (≤ 1.8U/L) and a positive result of IF total IgE (> 10.65 ng/ml) or IF/Serum IgE raito (> 0.48). All the patients had contact history with dogs and cats or lived in rural area. 8 of 9 patients (88.9%) presented with typical peripheral granuloma. The duration from disease onset to IF testing ranged from 1 month to 4 years. There were 3 patients in total with both positive results of IF total IgE and IF/Serum IgE ratio.

## Discussion

OT is one of the worldwide zoonotic infections which have the possibility of leading to blindness and has been neglected in many circumstances. Although unilateral granulomatous uveitis is typical of OT, the complicated clinical manifestations of OT make it hard definitely diagnosed. Previous studies reported the mean duration between the first symptoms and diagnosis could be as long as 15.1 months [12]. Similarly, in our study, the mean duration from disease onset to IF testing was nearly one year (Table 1). The delayed diagnosis seemed to be associated with higher rate of complications such as macular edema and retinal detachment thus resulting in worse vision prognosis [12, 13], which justified the need of rapid and accurate diagnosis methods. In fact, the specific IgG detection in serum and IF has been widely discussed in previous literature [5–8, 14–17]. The elevation of IF specific IgG level and GWC representing the local production of immunological antibodies could provide valid evidence for the diagnosis of OT to some extent. However, as the other important laboratory marker responding to parasite infection [11], IgE concentration change was little mentioned in the previous studies. Theoretically, local synthesis of IgE could also support the diagnosis of OT and the diagnostic value of IF total IgE deserves deeper research. In this study, we mainly investigated the diagnostic performances of IF total IgE level and IF/Serum IgE ratio in OT; Besides, considering the different immunological features of children and adults, we compared the IF IgE concentrations, IF/Serum IgE ratios and their diagnostic values in child OT and adult OT respectively. And the results turned out to be informative and enlightening.

First, in terms of the demographic and clinical features, our study found many facts similar to those reported by previous literature. OT was more prevalent in children with 68.4% of all the patients in this study. The percentage of male patients in child OT was significantly higher than that in adult OT (63.4% versus 33.3%, *p* = 0.014). In general, there are more male patients in OT ranging from 56.2–75.2% [12, 13, 18–22]. Only Hu et al.’s study reported fewer male patients in adult OT (45.8%). The difference of percentages of male patients might be due to the different sample sizes and different age groups. The possible reason of more male patients in child OT could be that boys are more likely to play with dogs and cats than girls especially in rural areas. Besides, poorer visual acuity was noted in child OT patients (Table 1). Since children usually couldn’t express the decrease of vision timely compared with adults, the clinical manifestations are more severe in child patients than adult patients, which could account for the poorer VA in children.

In current study, we found that IF IgE level and IF/Serum IgE ratio were both significantly higher in child OT patients than in adult patients. IgE is believed to contribute to host defense against certain parasites, particularly helminths [10]. In accordance with IgG, IgE is also long-standing and exists during the whole clinical course [23]. The level of local production of total IgE might vary at different stages of the disease. Despite no significant difference, the duration from disease onset to IF testing was longer in children, which meant the longer disease course of child OT patients. The above reasons might account for the higher IgE level noted in children. Besides, IF IgE level was higher in male patients than in female patients (Table 2) despite no significant difference after multiple comparisons, which could result from the bias of more male patients in child OT. Given the fact of higher IF IgE level in children, it’s reasonable to find higher IF IgE level in male patients as well. Previous studies [5, 8] reported VH samples showed increased local specific IgG and total IgE production compared with AH samples. Theoretically, OT is a clinical entity mainly involving the posterior segment of the eye, which could possibly lead to higher antibody production in vitreous than in AH. However, in this study, there is no significant difference of total IgE concentration in VH and AH after Holm–Bonferroni adjustment (*p* = 0.030 < 0.016) (Table 2). In addition, both IF IgE levels and IF/Serum IgE ratios of AH and VH showed good diagnostic performances with close AUCs despite higher cutoff values noted in VH (Table 5). In terms of the diagnosis of OT, Xu et al. [5] also reported that AH and VH samples had significant correlations and perfect agreements for both specific IgG and GWC, which was similar to our result of IF IgE level and IF/Serum IgE ratio.

In this study, we first investigated the diagnostic values of IF IgE level and IF/Serum IgE ratio in OT and confirmed the associated cutoff values. The best cutoff value of IF IgE level was 10.65 ng/ml yielding 77.6% sensitivity and 98.4% specificity. The high specificity might be due to the low level of IF IgE concentrations in control groups. Wang et al. reported the intraocular IgE production was found in 64/69 (92.75%) cases; However, they used the standard that the intraocular IgE levels were higher than the mean plus two SD value of normal IF control levels instead of a calculated cutoff value. When comparing the diagnostic performances between IF IgE level and IF specific IgG level, we found the latter was better than the former with larger AUC and higher sensitivity. The IF IgE level showed better diagnostic performance in child OT patients than in adult OT patients with higher sensitivity (82.7%) and similar specificity. This result was consistent with the fact that the IF IgE level was significantly higher in child OT patients that in adult OT patients. In terms of IF/Serum IgE ratio, the AUC of IF/Serum IgE ratio was significantly higher than that of GWC of IgG for OT. GWC must be combined with positive results of local IgG production to diagnose OT [7, 8] and the diagnostic value of plain GWC is limited. On the contrary, plain IF/Serum IgE ratio with cutoff value 0.48 could achieve 78.4% sensitivity and 98.4% specificity. For child OT, the cutoff value of IF/Serum IgE ratio for the diagnosis of child OT was 0.52, close to the standard (0.60) mentioned in Wang et al.’s study [8]. Given the higher IF IgE level in children, the cutoff value of IF/Serum IgE ratio was higher than the 0.48 for all OT patients.

Instead of using the cutoff value (1.01U/L) of IF IgG calculated in the current study, we chose the cutoff value of 1.8 U/L reported by Huang et al.’s study [7] considering the larger sample size in their study. Table 5 shows there are 9 patients with a negative result of IF specific IgG (≤ 1.8U/L) and a positive result of IF total IgE (> 10.65 ng/ml) or IF/Serum IgE raito (> 0.48). The possible reason might be due to the earlier seroconversion of IgE than specifc IgG and different assay methods. The above results demonstrated that the test of intraocular IgE concentration was meaningful and combining the indicators of total IgE and specific IgG might be helpful to improve the sensitivity for the diagnosis of OT in a sense.

There are several limitations in this study. Firstly, the control group with non-OT uveitis displayed relatively low IgE levels, which might result in lower cutoff values for diagnosis. Secondly, the subtypes of OT were not clearly separated for analysis in this study and we didn’t investigate the differences of total IgE concentrations in different clinical forms of OT. Thirdly, the role of intraocular IgE level monitoring the disease course during follow-up was not discussed and further study is needed for investigating the changes of IgE level during the different stages of the disease. Last but not the least, this study was limited as a single-center retrospective study and the sample size was relatively small especially for the adult OT patients, which could induce bias for analysis to some extent. Therefore, a prospective multicenter study with larger sample size was required for future work and avidity tests [24, 25] of IgE and IgG might be employed to represent the disease course more precisely.

## Conclusions

In summary, our study is the first to evaluate the diagnostic values of the intraocular total IgE level and its ratio with serum IgE level for OT and put forward the corresponding cutoff values for further use. We found both IF IgE level and IF/Serum IgE ratio demonstrated good diagnostic performance for OT and higher sensitivity of the IF total IgE level was noted for the diagnosis of child OT.

## Data Availability

The datasets used and/or analyzed during the current study are available from the corresponding author on reasonable request.

## Abbreviations

OT: Ocular Toxocariasis
IF: Intraocular Fluid
GWC: Goldmann-Witmer Coefficient
AH: Aqueous humor
VH: Vitreous humor
AUC: Area under the curve
VA: Visual Acuity
CI: Confidence Interval
